# Moving forward through the in silico modeling of tuberculosis: a further step with UISS-TB

**DOI:** 10.1186/s12859-020-03762-5

**Published:** 2020-12-14

**Authors:** Giulia Russo, Giuseppe Sgroi, Giuseppe Alessandro Parasiliti Palumbo, Marzio Pennisi, Miguel A. Juarez, Pere-Joan Cardona, Santo Motta, Kenneth B. Walker, Epifanio Fichera, Marco Viceconti, Francesco Pappalardo

**Affiliations:** 1 Department of Drug Sciences, University of Catania, 95125 Catania, Italy; 2 Department of Mathematics and Computer Science, University of Catania, 95125 Catania, Italy; 3 Computer Science Institute, DiSIT, University of Eastern Piedmont, 15121 Alessandria, Italy; 4 School of Mathematics and Statistics, University of Sheffield, Sheffield, S3 7RH UK; 5 Archivel Farma, S.L., 08916 Badalona, Spain; 6 Experimental Tuberculosis Unit (UTE), Fundació Institut Germans Trias I Pujol (IGTP), Universitat Autònoma de Barcelona (UAB), Badalona, Spain; 7 Centro de Investigación Biomédica en Red (CIBER) de Enfermedades Respiratorias, Madrid, Spain; 8 National Research Council of Italy, 00185 Rome, Italy; 9 TuBerculosis Vaccine Initiative (TBVI), Lelystad, 8219 The Netherlands; 10 Etna Biotech S.r.l., 95121 Catania, Italy; 11 Department of Industrial Engineering, University of Bologna, 40136 Bologna, Italy

**Keywords:** Tuberculosis, Computational modeling, In silico trials, RUTI, Isoniazid, Immunity, Therapeutic strategies

## Abstract

**Background:**

In 2018, about 10 million people were found infected by tuberculosis, with approximately 1.2 million deaths worldwide. Despite these numbers have been relatively stable in recent years, tuberculosis is still considered one of the top 10 deadliest diseases worldwide. Over the years, Mycobacterium tuberculosis has developed a form of resistance to first-line tuberculosis treatments, specifically to isoniazid, leading to multi-drug-resistant tuberculosis. In this context, the EU and Indian DBT funded project STriTuVaD—In Silico Trial for Tuberculosis Vaccine Development—is supporting the identification of new interventional strategies against tuberculosis thanks to the use of Universal Immune System Simulator (UISS), a computational framework capable of predicting the immunity induced by specific drugs such as therapeutic vaccines and antibiotics.

**Results:**

Here, we present how UISS accurately simulates tuberculosis dynamics and its interaction within the immune system, and how it predicts the efficacy of the combined action of isoniazid and RUTI vaccine in a specific digital population cohort. Specifically, we simulated two groups of 100 digital patients. The first group was treated with isoniazid only, while the second one was treated with the combination of RUTI vaccine and isoniazid, according to the dosage strategy described in the clinical trial design. UISS-TB shows to be in good agreement with clinical trial results suggesting that RUTI vaccine may favor a partial recover of infected lung tissue.

**Conclusions:**

In silico trials innovations represent a powerful pipeline for the prediction of the effects of specific therapeutic strategies and related clinical outcomes. Here, we present a further step in UISS framework implementation. Specifically, we found that the simulated mechanism of action of RUTI and INH are in good alignment with the results coming from past clinical phase IIa trials.

## Background

Tuberculosis (TB), a disease caused by Mycobacterium tuberculosis (MTB) infection, is still one of the top 10 causes of death worldwide, especially in emerging countries. According to the World Health Organization (WHO), in 2018 10 million people fell ill with tuberculosis, of which 5.7 million were men, 3.2 million women, and 1.1 million children [1]. TB spreads from person to person through aerosol transmission. As today, no one is immune or isolated from the risk of being affected by the disease [2], and no prophylactic vaccines are available. Recently, bedaquiline and delamanid were approved as new two anti‐TB drugs, while novel candidates and repurposed drug have been developed and are in the final stages of drug development process [3].

Commonly, first-line TB treatments (isoniazid (INH), rifampin (RIF), pyrazinamide (PZA), ethambutol (EMB) and streptomycin (SM) [4]) are used for active tuberculosis in order to reduce the bacterial load in the lungs and the probability of transmission. INH, already known as isonicotinic acid hydrazide [5], is one of the leading standard antibiotic treatments for people at low risk for drug-resistance, leading to a significant reduction of mycobacterial load [6]. INH inhibits the synthesis of mycolic acids, an essential component of the bacterial cell wall, and is used in conjunction with other effective anti-tuberculosis agents in a multi-drug therapy protocol [7]. INH has a short half-life ranging from 1 to 4 h [8] and a double-activity: for the first 24 h from the administration, INH shows a bacteriostatic mechanism, then its activity becomes bactericidal [9–11]. This pro-drug requires a preliminary activation, which is carried out by the heme enzyme catalase/peroxidase (KatG) of MTB [12]. The interactions between the host immune system and INH allow to decrease the growth of intracellular and extracellular MTB bacilli. It is worth mentioning that the active form of tuberculosis should be treated with different combinations of anti-tuberculosis drugs to prevent the emergence of drug resistance phenomena. This is due to the fact that the single use of isoniazid for active tuberculosis is not always effective. The high bactericidal activity, the elevated intracellular penetration, and the low costs make INH one of the most commonly used antimicrobial agents to fight tuberculosis.

Second-line drugs are sub-divided into two categories: fluoroquinolones (ofloxacin (OFX), levofloxacin (LEV), moxifloxacin (MOX) and ciprofloxacin (CIP) and injectable antituberculosis drugs (kanamycin (KAN), amikacin (AMK) and capreomycin (CAP). Other second-line antituberculosis (ethionamide (ETH)/prothionamide (PTH), cycloserine (CS)/terizidone, p-aminosalicylic acid (PAS) [4]) are used for the treatment of drug-resistant and multi-drug-resistant TB patients. However, second-line treatment options own some disadvantages because they require very long treatment regimens, along with a toxic exposure and high costs for the patients.

To overcome these issues, many EU-funded projects are trying to fight TB with specific trials concerning new therapeutic strategies. Among these, the HORIZON 2020 In Silico Trial for Tuberculosis Vaccine Development (STriTuVaD) project aims to evaluate a specific therapeutic vaccine against tuberculosis through an innovative computational modelling infrastructure named Universal Immune System Simulator (UISS). In this context, UISS for Tuberculosis (UISS-TB) is capable to reproduce the dynamics of the immune system affected by TB and predict the outcome of a real clinical trial under the administration of specific interventions such as the RUTI vaccine [13].

RUTI vaccine is a polyantigenic liposomal vaccine previously used as an immunotherapeutic intervention during antibiotic treatment for Multi-Drug-Resistant Tuberculosis (MDR-TB). This vaccine showed an excellent safety profile in a phase 2 trial [14], and has the potential to reduce the time required for the current antibiotic treatments significantly.

To investigate the effects of the combination of a conventional anti-TB chemotherapy strategy with a potential therapeutic vaccine such as RUTI, we present here an extension of our pre-existing version of UISS-TB able to predict their combined efficacy.

## Methods

UISS computational framework, widely discussed in [13], was successfully applied to a large number of disease modelling scenarios [15–17], including COVID-19 [18]. UISS is based on Agent-Based Model (ABM) methodology [19, 20] that predicts the efficacy of vaccines and/or antibiotics treatments targeting MTB in a specific digital patients cohort.

To simulate the effects of isoniazid, we implemented in UISS its mechanism of action and modeled the effects on MTB using the administration protocol described in [14] for one month.

To this end, we added INH as a new entity (also called “agent” in ABM terminology) into the simulation framework. INH agents are described through their concentration in each position of simulation space and their half-life, used by the simulation framework to calculate the degradation of INH and to manage the bacteriostatic and bactericidal activities. INH injection time and quantities are defined according to the administration protocol described in [14]. The interaction that describes the effects of INH against MTB can be briefly reassumed as follows. The bacteriostatic effect was implemented considering infected AM cells that encounter INH formulation within the lung compartment. With a probability dependent on INH concentration, the intracellular and extracellular replication rates of MTB are reduced. The mathematical law that describes the intracellular and extracellular proliferation of MTB has been implemented with a method similar to the one described in [21, 22]. The bactericidal activity of INH affects the circulating TB bacilli. With a probability depending on INH concentration, circulating TB bacilli are cleared from the bloodstream.

Moreover, we also revised the RUTI implementation in UISS, to better model the mechanism of action (MoA) and the vaccine interaction with the host immune system. According to RUTI formulation, we added the liposome entity and its related dynamics. At the beginning of each simulation, the user provides UISS with two parameters: i) RUTI dosage and ii) the time of the administration. The aim was to allow UISS to simulate the interaction between the liposome and dendritic cell (DC). In particular, when in the lymph node compartment, a naïve DC encounters a liposome, DC scans the surface of the liposome to recognize the MHC-1/peptide complex. Through the calculation of a proportional probability function that depends on the affinity level between DC pattern recognition receptor (DC-PRR) and MHC-1/peptide complex expressed on the surface of the liposome. This immunological process led by DC is known as nibbling. Finally, the liposome—CD8 T cells (TC) interaction has been added. If the interaction succeeds, i.e., TC recognizes the MHC-I/liposome peptide on the liposome surface (by affinity score calculation), the TC releases a predefined quantity of interferon gamma (IFN-γ) in situ. The release of free antigens through the liposomes (due to physiological degradation) over time has also been considered.

To better represent the biological diversity of TB patients, we enriched the composition of the vector of features used for the generation of digital patients libraries. In particular, the "vector of features" that defines a specific TB patient is composed by the following parameters: (1) MTB virulence; (2) MTB Sputum; (3) CD4-Th1; (4) CD4-Th2; (5) IgG; (6) TC; (7) IL-23; (8) IL-12; (9) IL17-A; (10) IL-2; (11) IL-1; (12) IL-10; (13) IFN1A; (14) IFN1B; (15) IFNG; (16) TNF; (17) Treg; (18) LXA4; (19) PGE2; (20) Vitamin D; (21) Age; (22) BMI. The digital patients were generated according to the steps explained in [23]. Table 1 summarizes the biological description of each entity that composes the vector of features, specifying the unit of measurements and the values used to run the simulations.

**Table 1:** Vector of features

#	Parameter name	Unit of measurement	Biological description	Values set in the simulations
1	MTB virulence	Real	The ability of a mycobacteria to cause tuberculosis, depending on its capability to reside within host cells and evade the microbicidal mechanisms of macrophages	0.5
2	Mtb sputum	CFU/mL	Mycobacterium bacilli present in sputum smear traditionally quantified by counting colony forming units	80,000
3	CD4^+^-Th1	cells/µL	Lineage of CD4^+^ effector T cell required for host defense against pathogens promoting cell-mediated immune responses	0
4	CD4^+^-Th2	cells/µL	Lineage of CD4^+^ effector T cell required for humoral immunity promoting the coordination of the immune response to extracellular pathogens	0
5	IgG	titer	The main type of antibody in blood and extracellular fluid, allowing control infection of body tissues and body protection from intracellular caused infections	0
6	TC (CD8^+^)	cells/µL	A type of lymphocyte that can kill foreign cells, cancer cells, and cells infected with a virus	562
7	IL-23	pg/mL	A proinflammatory cytokine involved in the induction of IL-17-producing antigen-specific CD4 + T cells (Th17) and in the control of tuberculosis. It also outlines the expression of vaccine-induced protection against tuberculosis	0
8	IL-12	pg/mL	A proinflammatory cytokine naturally produced by dendritic cells, macrophages, neutrophils in response to antigenic stimulation	0
9	IL17-A	pg/mL	A proinflammatory cytokine produced by activated T helper cells in response to their stimulation with IL-23. In tuberculosis, it represents a protective cytokine against mycobacteria	0
10	IL-2	pg/mL	A proinflammatory cytokine that stimulates the growth and replication of B lymphocytes (B cells) and T lymphocytes (T cells). It is significantly higher in active TB patients, suggesting that IL-2 represents a potential infection severity biomarker	0
11	IL-1	pg/mL	An anti-inflammatory cytokine produced by macrophages. It usually raises body temperature, spurs the production of interferon, and stimulates growth of disease-fighting cells. IL-1 receptor pathways are essential for the control of MTB infection	0
12	IL-10	pg/mL	An anti-inflammatory cytokine with multiple, pleiotropic, effects in immunoregulation and inflammation. It downregulates the expression of Th1 cytokines, MHC class II antigens, and co-stimulatory molecules on macrophages. It also enhances B cell survival, proliferation, and IgE antibody production. IL-10 has been identified as a correlate of susceptibility for tuberculosis and reactivation of TB disease	0
13	IFN1A	pg/mL	Human type I interferons (IFNs) are a large subgroup of interferon proteins that help regulate the activity of the immune system. The IFN-α proteins are produced mainly by plasmacytoid dendritic cells (pDCs). They are mainly involved in innate immunity against viral infection	0
14	IFN1B	pg/mL	Human type I interferons (IFNs) are a large subgroup of interferon proteins that help regulate the activity of the immune system. The IFN-β proteins are produced in large quantities by fibroblasts. They have antiviral activity that is involved mainly in innate immune response	0
15	IFNG	pg/mL	A proinflammatory cytokine primarily secreted by activated T cells and natural killer (NK) cells that promote macrophage activation, mediate antiviral and antibacterial immunity, enhance antigen presentation, orchestrate activation of the innate immune system, coordinate lymphocyte–endothelium interaction, regulate Th1/Th2 balance, and control cellular proliferation and apoptosis. It represents the clinical standard that establish the evidence of Mtb exposure and infection	0
16	TNF	pg/mL	N inflammatory cytokine produced chiefly by activated macrophages and many other cell types such as T helper cells, natural killer cells, neutrophils, mast cells, eosinophils, and neurons. Itplays a major role in the initial and long-term control of tuberculosis	0
17	Treg	cells/µL	T cells which have a role in regulating or suppressing other cells in the immune system. Tregs control the immune response to self and antigens and help prevent autoimmune disease	68
18	LXA4	ng/mL	A bioactive autacoid metabolite of arachidonic acid that displays both potent anti-inflammatory and pro-resolving actions. In tuberculosis disease, it owns a pro-necrotic activity against infected alveolar macrophages	0
19	PGE2	ng/mL	A lipid compounds called eicosanoids having several hormone-like effects in animals. It derives enzymatically from the fatty acid arachidonic acid. In tuberculosis disease, it owns a pro-apoptotic activity against infected alveolar macrophages	0
20	VitaminD	ng/mL	It is considered an essential micronutrient involved in several biological processes such as endocrine metabolism and immune system activity, by modulating and inhibiting its activity in different ways. Its deficiency is associated with the risk of tuberculosis infection	25.8
21	AGE	years	A risk factor that should be considered for tuberculosis incidence and prognosis	35
22	BMI	kg/m^2^	A key index for relating weight to height. BMI has been found correlated with both active and latent forms of tuberculosis	21

Simulator input parameters that compose the vector of feature to personalize the digital patient. A biological description along with the values used to run the simulations are provided as well

**Fig. 1:**
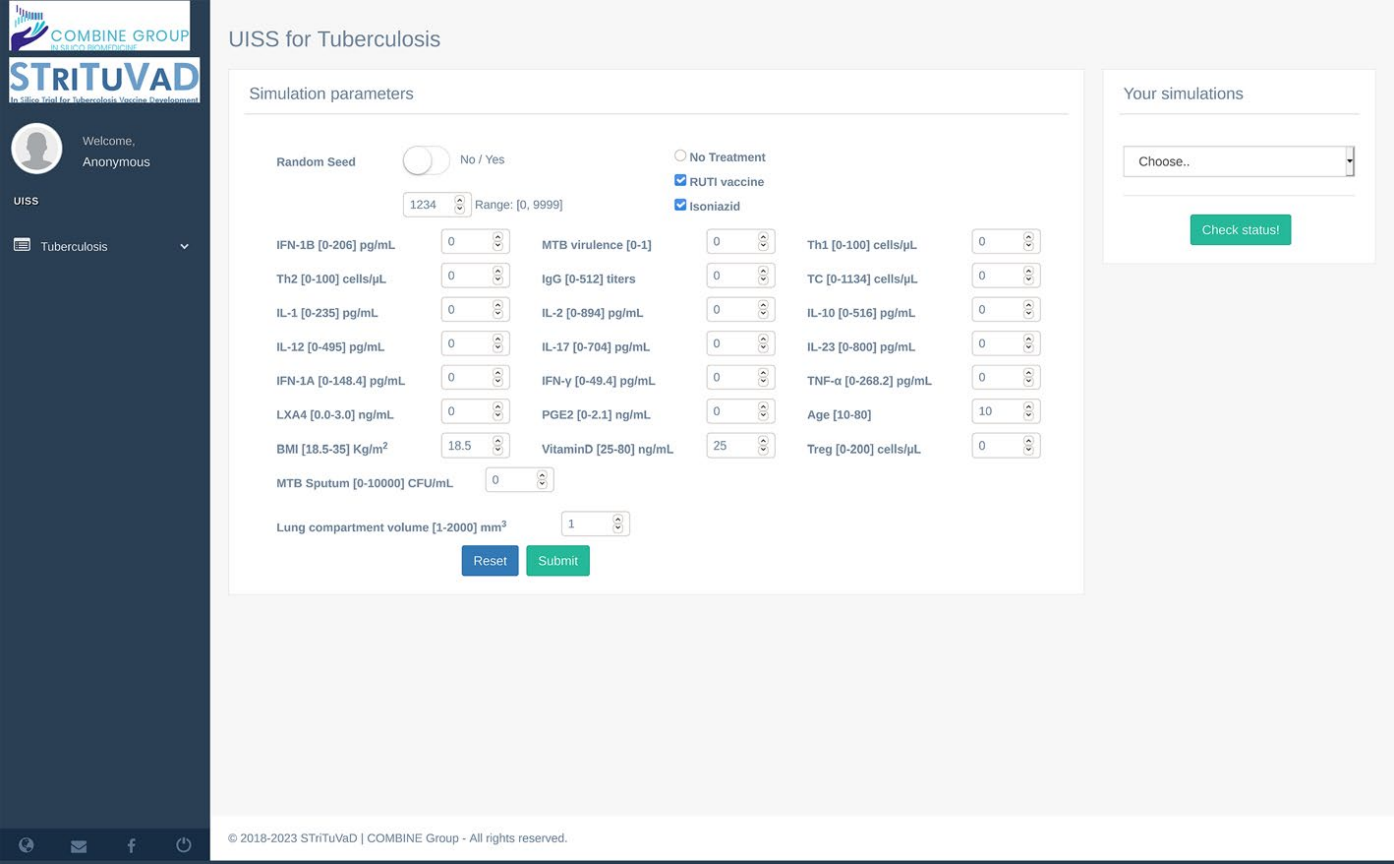
Web Graphic User Interface of UISS-TB. This figure depicts the GUI of UISS that allows the run of the simulations. The "Simulation's Parameters" zone, on the left side of the figure represents the vector of features for the personalization of digital patients. The "Your Simulations" box, on the right side of the figure, depicts the list of all the simulations launched by the user. The simulations are classified in "running" or in "completed" status

As UISS is written in C language, a Graphic User Interface (GUI) and a web server is needed to provide a user-friendly interface. In a previous work, we presented a web-interface developed in Flask micro-server [24]. Here, we improved the performance of the web platform. These enhancements allow the launch of the simulations separately from the main thread and in a more efficient way. To this aim, we used Django, the high-level Python Web framework. Figure 1 shows the last version of the UISS web-GUI. On the right side, one can see a box called "*Your simulation*" containing a list of the simulations, sorted by their creation date and classified in "running" or in "completed" status. On the left side, one can see a box named "*Simulation Parameters*" that contains a set of the biological and physiopathological parameters that compose the vector of features, created for the customization of TB patients.

In details, after the user connects to the UISS-TB web interface, she/he selects the Tuberculosis disease model. After that, the general GUI panel appears. The user finds already filled in default values in the vector of features parameters. She/he can vary these values according to the ranges that are shown within brackets, near the selected parameter. After that, one can press the Submit button and a unique identification simulation number is assigned. The user can check the simulation status simply clicking on the check status button, after selecting the simulation id. When the simulation is completed, the user can visualise results of immune system dynamics, simply choosing the one she/he would like to analyze.

## Results and discussion

A first step in moving UISS towards clinical validation was to evaluate its prediction capabilities. In this context, we designed different simulations over cohorts of digital patients to obtain accurate in silico predictions about the efficacy of therapeutic interventions directed against TB. We run a total of 400 simulations, within four different scenarios: (1) 100 digital patients treated with INH only; (2) 100 digital patients treated with the combination of INH and RUTI vaccine; (3) 100 digital patients treated with the combination of INH and two RUTI vaccine administrations; (4) 100 digital patients treated with the combination of INH and two RUTI vaccine administrations, at different timing. We took into account only drug-sensitive digital patients (i.e., not affected by MDR-TB). MTB infection peaks after two weeks from the starting of the simulation.

**Fig. 2:**
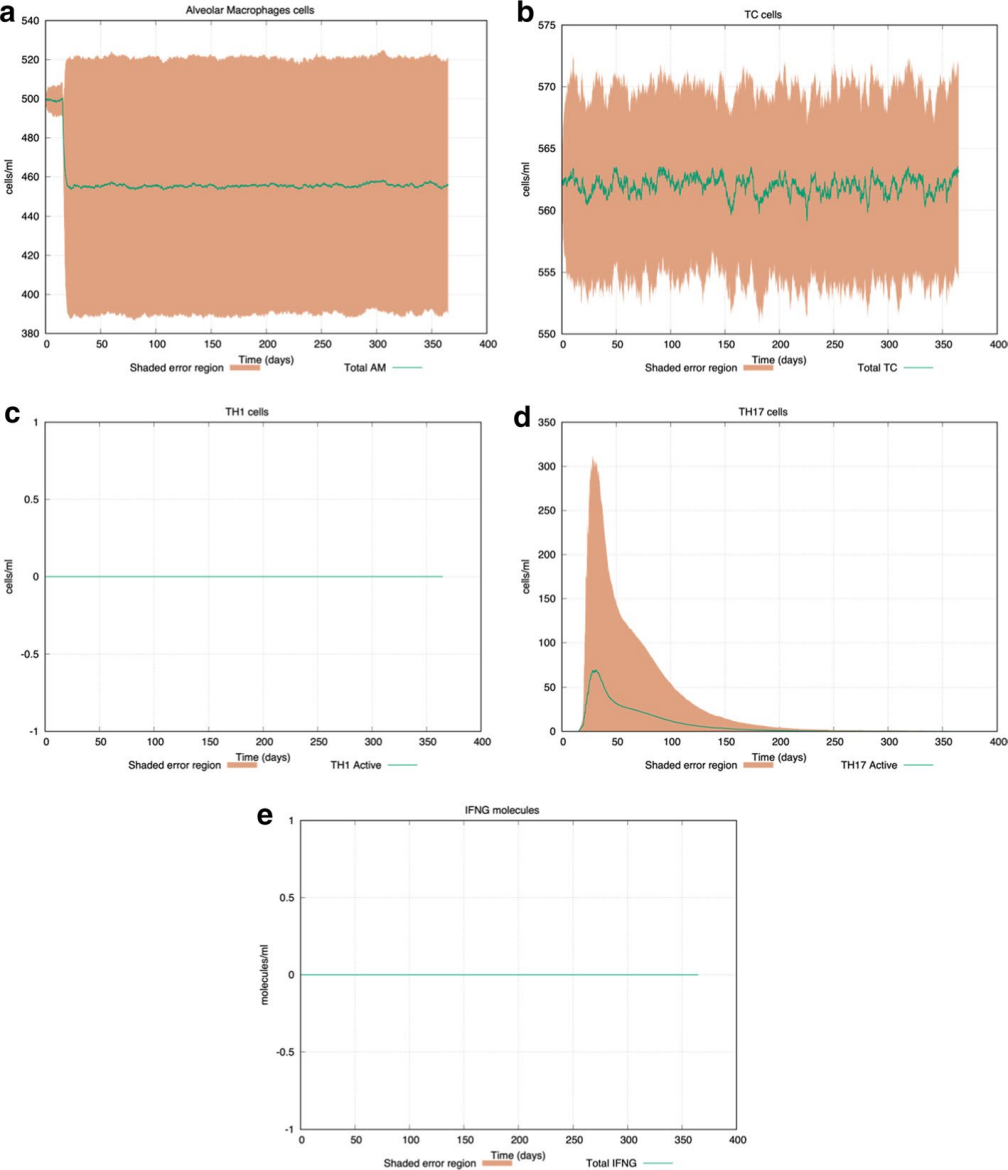
Outcome of digital patients treated with INH. Green line shows the average trend of the considered cellular entities. The orange shaded area represents their standard deviation (SD + /−). **a** Depicts the dynamics of AM before and after the administration of INH; the antibiotic, administered accordingly to the clinical trial protocol, reveals a not negligible biological restore of the damaged AM. **b** The dynamics of CD8 T cells. **d** The dynamics of TH17 cells responding to bacterial infection. **c** and **e** show flat curves because INH is not supposed to stimulate immune response. Simulation time has been set to 365 days (1 years) and digital patients have been challenged with MTB at day 15

**Fig. 3:**
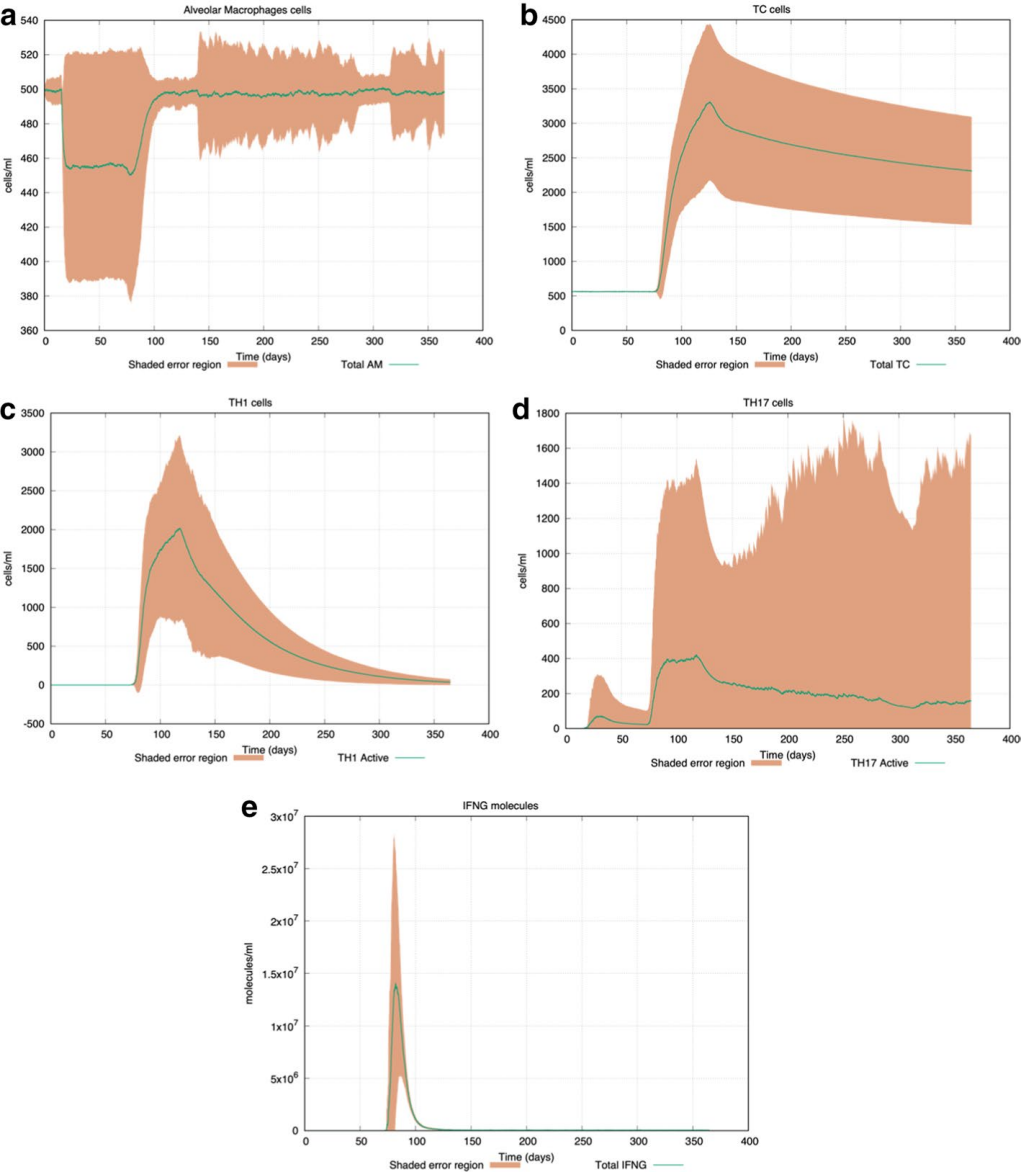
Outcome of digital patients treated with INH and RUTI vaccine. Green line shows the average trend of cell populations, while the orange shaded area represents the standard deviation. One month after the end of antibiotic treatment, RUTI vaccine was administered accordingly to the clinical trial design. **a** Depicts the dynamics of AM before and after the administration of INH and after the administration of RUTI. The combination of INH with RUTI allows a better recovery of infected AM population when compared to the one without RUTI injection. Substantial increase in levels of TC, Th1, Th17 and IFNG is observed (**b**–**e**). For all the biological scenarios, simulation time has been set to 365 days (1 years) and digital patient have been challenged with MTB at day 15

In order to show the effects of RUTI vaccination protocol complemented with the administration of INH on the host immune system, we tracked the dynamics of Alveolar Macrophages (AM), CD4 Th1, Interferon-gamma (IFN-γ), Cytotoxic T cells (TC), CD4 Th17. In Figs. 2 and 3, the mean behavior (green line) and standard deviation (orange shaded region) of the biological entities taken into consideration are depicted. Untreated TB digital patients have been widely discussed in [13, 24]. Figure 2 shows the cellular dynamics where an initial challenge with a virulent strain of MTB is supposed to happen on day 15. Soon after, we simulated the injection of INH once a day for one month. Figure 2, panel A shows the dynamics of AM; here, we can observe a not negligible reduction of the average population of necrotic AM. In other words, the injection of INH allows a tissue recovery of the lung's patient. Regarding cytotoxic CD8 T cells, Fig. 2, panel B shows no significant differences from the untreated cases. This is in good agreement with clinical observations as INH antibiotic therapy does not affect immune system behavior. Accordingly, panel C and panel E of Fig. 2 shows no Th1 cell activation and no IFN-γ presence [25].

Th17 cells are a subpopulation of helper T cells. Their production is stimulated by cytokines such as IL-6, IL-1, and IL-23, produced in response to extracellular bacteria, such as in tuberculosis [25]. They are involved in the recruitment of leukocytes to the infection site and have an essential role in the elimination of bacteria. Figure 2 panel D, depicts the increase of Th17 cells in response to MTB infection. After an initial burst, Th17 number gradually reduces as patients convert into latent tuberculosis [26].

Figure 3 shows the second scenario in which the administration of RUTI vaccine is coupled with INH. As above, we kept the MTB challenge on day 15, followed by INH administration (once a day per one month). This time, one month after the end of the antibiotic treatment, an injection of 25 μg of RUTI vaccine was simulated.

Panel A, Fig. 3 depicts the dynamics of AM. In this case, one can see as RUTI significantly reduces the AM necrotic population. According to literature, panel B of Fig. 3 highlights an increased activation of CD8^+^ T cells. The diminution of CD8^+^ T cells in the latent stage of the infection led to an increase in the bacterial load, which indicates that these cells are necessary for the long-term control of the disease [27]. This could suggest that a second RUTI administration could be beneficial to the MTB eradication. Figure 3 panel C shows a considerable Th1 response that is also supported by a not negligible release of IFN-γ (panel E). There is also an increased activation of Th17 (panel D). Reassuming, the RUTI orchestrated immune response is in very good agreement with specialized literature [14].

**Fig. 4:**
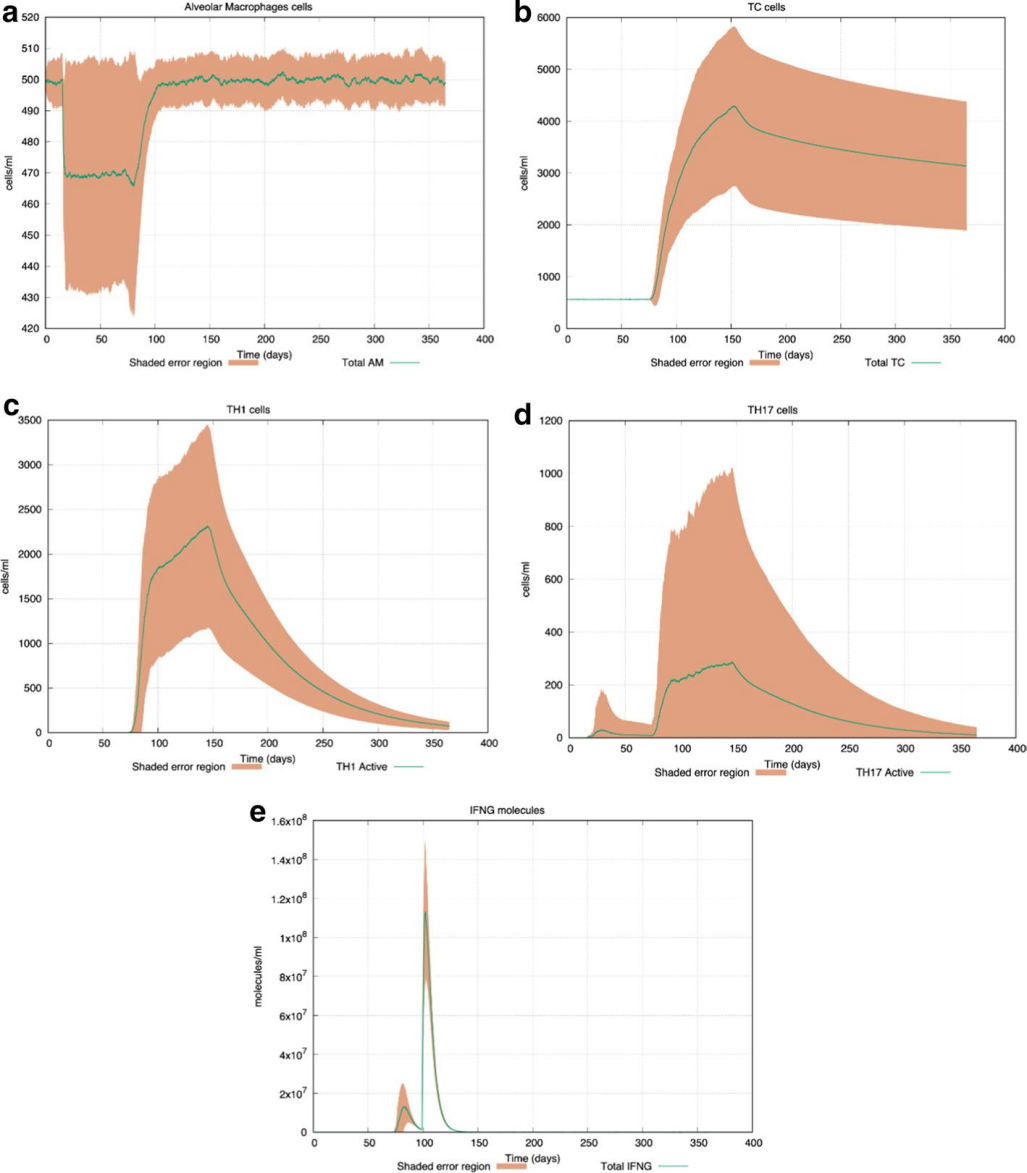
Outcome of digital patients treated with the second RUTI vaccine administration. Green line shows the average trend of cell populations, while the orange shaded area represents the standard deviation. One month after the end of antibiotic treatment, RUTI vaccine was administered accordingly to the clinical trial design followed by a second injection of RUTI (28 days after the first one). **a** The dynamics of AM that is comparable to the scenario observed after only one vaccine administration. Substantial increase in levels of TC, Th1, Th17 and IFNG is observed (**b**–**e**) compared to dynamics obtained with only one vaccine administration. For all the biological scenarios, simulation time has been set to 365 days (1 years) and digital patient have been challenged with MTB at day 15

Figure 4 shows the results of the second RUTI injection as reported in the clinical protocol design [28]. In comparison to the immune response obtained with one RUTI administration as previously shown in Fig. 3, here one notices a stronger CD4^+^ Th1 response (panel C) followed by an increased IFN- γ levels (panel E). Moreover, also CD8^+^ T cell response (panel B) is positively triggered by the second administration of RUTI.

**Fig. 5:**
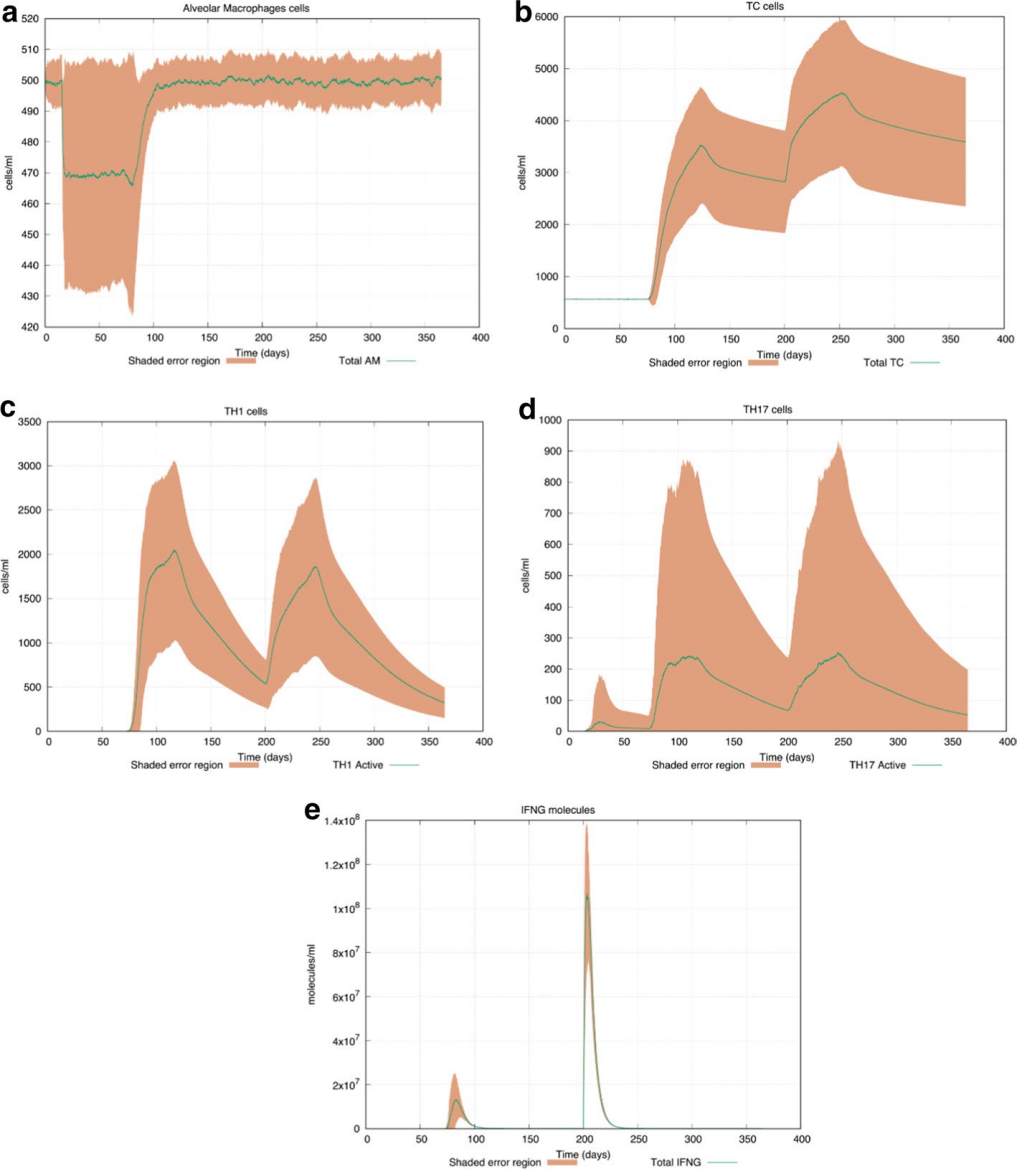
UISS in silico predictions with different timing of a second RUTI vaccine administration. Green line shows the average trend of cell populations, while the orange shaded area represents the standard deviation. In comparison to the scenarios observed in Fig. 4, a negligible difference in the overall immune response driven by CD4^+^ Th1 cells and CD8^+^ T cells is observed

To assess if a different timing of a second RUTI injection could improve the overall immune response of the host against MTB, we simulated a later second injection time compared to the clinical trial protocol design (i.e., at day 200, about three months after the one set in the clinical trial). In Fig. 5, we report the in silico predictions of such a different timing of second RUTI administration. A negligible difference in the overall immune response driven by CD4^+^ Th1 cells and CD8^+^ T cells is observed. This suggests that the timing agreed in the clinical trial dossier corresponds to the optimal one.

## Conclusions

In silico trials are increasingly used to predict the effects of several types of interventional strategies and related clinical outcomes. In this context, the EC and Indian DBT funded project STriTuVaD aims to create a computational infrastructure that predicts the efficacy of antibiotic strategies when coupled with RUTI vaccine against M. tuberculosis. Here, we present a further step in UISS framework implementation toward the clinical validation of the platform. We found that the simulated MoA of RUTI and INH is in proper alignment with the double-blind, randomized, placebo-controlled phase II clinical trial. To explore potential possibilities to increase the overall immune response against MTB, we simulated 100 digital patients treated with INH and two RUTI vaccine administrations varying the timing of the second one. In silico results confirm that the overall immune response driven by CD4^+^ Th1 cells and CD8^+^ T cells is not influenced, suggesting that the timing agreed in the clinical trial protocol is optimal.

## Availability and requirements

*Project name* UISS-TB.

*Project home page* https://www.combine-group.org/software.

*Operating system(s)* Platform independent.

*Programming language* C and Python.

*Other requirements* none.

*Any restrictions to use by non-academics* not applicable.

## Data Availability

The main computational framework is fully described in the paper. The UISS-TB framework used for this research is available at: https://combine.dmi.unict.it/UISS-TB/.

## Abbreviations

TB: Tuberculosis
MTB: Mycobacterium tuberculosis
STriTuVaD: In Silico Trial for Tuberculosis Vaccine Development
UISS: Universal Immune System Simulator
INH: Isoniazid
MDR-TB: Multidrug-resistant tuberculosis
WHO: World Health Organization
ABM: Agent-based modeling
KatG: Heme enzyme catalase/peroxidase
MHC-I: Major histocompatibility complex class I
MHC-II: Major histocompatibility complex class II
APC: Antigen processing cell
MoA: Mechanism of action
IgG: Immunoglobulin class G
AM: Alveolar macrophage
N: Neutrophils
LXA4: Lipoxin A4
PGE2: Prostaglandin E2
IFN-γ: Interferon-gamma
TReg: Regulatory T cells
TC: Cytotoxic T cells
TNF: Tumor necrosis factor
GUI: Graphic user interface
